# An Update on the Key Factors Required for Plant Golgi Structure Maintenance

**DOI:** 10.3389/fpls.2022.933283

**Published:** 2022-06-28

**Authors:** Qingchen Rui, Xiaoyun Tan, Feng Liu, Yiqun Bao

**Affiliations:** College of Life Sciences, Nanjing Agricultural University, Nanjing, China

**Keywords:** Golgi stack, Golgi structure, intra-Golgi trafficking, COPI, trafficking machinery

## Abstract

Plant Golgi apparatus serves as the central station of the secretory pathway and is the site where protein modification and cell wall matrix polysaccharides synthesis occur. The polarized and stacked cisternal structure is a prerequisite for Golgi function. Our understanding of Golgi structure maintenance and trafficking are largely obtained from mammals and yeast, yet, plant Golgi has many different aspects. In this review, we summarize the key players in Golgi maintenance demonstrated by genetic studies in plants, which function in ER-Golgi, intra-Golgi and post-Golgi transport pathways. Among these, we emphasize on players in intra-Golgi trafficking.

## Introduction

The secretory pathway is an essential vesicle trafficking process in eukaryotic cells. Golgi apparatus occupies a central position of the pathway, receiving *de novo* synthesized molecules from the endoplasmic reticulum (ER), carrying on glycan remodeling and other modification on biosynthetic cargo proteins, subsequently, cargo are sorted at the *trans*-Golgi network (TGN) and exported to their ultimate destination, the plasma membrane (PM), or the lysosome/vacuole. The plant Golgi apparatus plays a critical role in cell wall biosynthesis as it is the site to produce cell wall matrix polysaccharides, such as hemicelluloses and pectins, and to assemble/transport of cellulose synthase (CESA) complexes (CSCs; Glick and Nakano, 2009; Ito et al., 2014; Robinson, 2020).

An individual Golgi stack in most eukaryote cells adopts a unique polar structure where five to eight flattened membrane discs called cisternae layered on top of each other in a *cis* to *trans* direction, with *cis*-cisternae facing the ER. Despite this similarity, the Golgi bodies in plant cell are organized differently from the animal ones. While dozens of laterally connected Golgi stacks form a perinuclear immobile ribbon in animal cells (Lowe, 2011; Saraste and Prydz, 2019), the Golgi apparatus in plant cells consists of discrete, highly mobile Golgi stacks traveling throughout the cytoplasm on actin filaments (Boevink et al., 1998). In the presence of a large vacuole occupies 95% of the volume of most plant cell types, these mobile Golgi bodies are flexible to capture cargo from ER and send them to the downstream compartments. Unlike in mammalian cells, where Golgi apparatus disassembled during cell cycle, plant Golgi stacks remain intact and present in close proximity to the phragmoplast, so that Golgi-derived vesicles can supply new materials to the cell plate (Nebenfuhr et al., 2000). Another distinct characteristic of plant Golgi is that TGN is a comparatively independent compartment that also functions as the early endosome (EE; Dettmer et al., 2006; Lam et al., 2007; Viotti et al., 2010). The distinct features of plant Golgi facilitate the execution of its specific functions such as building of the cell plate during mitosis, synthesizing cell wall components or fast releasing of immune molecules by focally re-localizing toward the pathogen attacking sites (Ito et al., 2014; Yun and Kwon, 2017).

In mammalian cells, there is long-lasting debate on two predominant models of secretory cargo progression through the Golgi stack: cisternal maturation versus vesicular transport (Pelham and Rothman, 2000; Glick and Nakano, 2009; Boncompain and Weigel, 2018). Briefly, in the cisternal maturation model, cisternae are transient compartments out growing from the ER, carrying the secretory cargo forward and maturing over time through recycling of resident Golgi proteins to younger cisternae *via* retrograde COPI (coat protein I) vesicles. Cargo proteins reach the TGN and exit by clathrin-coated vesicles or other carriers. By contrast, in the vesicular transport model, the Golgi is composed of relatively stable cisternae, each of which contains a unique set of Golgi resident enzymes. Anterograde COPI vesicles carry secretory cargo forward, while exclude Golgi resident proteins. Studies from *Saccharomyces cerevisiae* are in favor of the cisternal maturation model (Losev et al., 2006; Matsuura-Tokita et al., 2006) whose Golgi apparatus is composed of individual *cis-*, *medial-*, and *trans-*cisternae scattered in the cytoplasm (Preuss et al., 1992). Recently, it is further supported in *S. cerevisiae* by high-resolution confocal observation utilizing three different fluorescent markers (to label the transported cargo, *cis-* and *trans*-cisternae, respectively), where secretory cargo remains in the same cisterna during *cis* to *trans* maturation (Kurokawa et al., 2019). The cisternal progression/maturation model of plant Golgi has also been supported (Robinson, 2020; Kang et al., 2022; see the section “Post-Golgi Trafficking”). In mammalian cells, other transport model is also proposed where transient inter-cisternal tubules are formed to permit the rapid diffusion of some cargo across the Golgi stack (San Pietro et al., 2009; Yang et al., 2011; Beznoussenko et al., 2014; Park et al., 2015).

**Figure 1:**
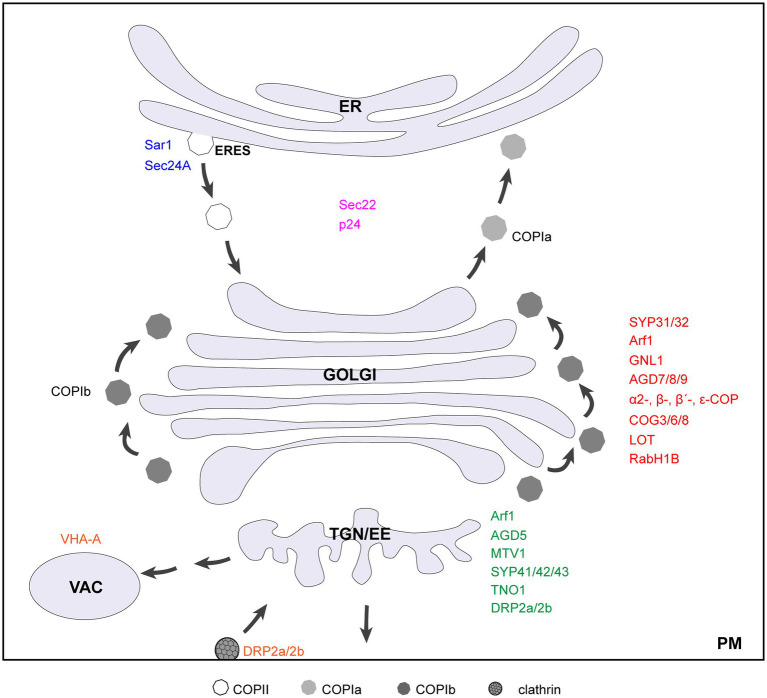
Diagram of a cell showing key genetic elements involved in plant Golgi structure maintenance. Factors that localize to ERES, Golgi, TGN, and vacuole/PM are indicated in blue, red, green, and orange, respectively. The proteins recycle between ER and Golgi are indicated in purple. Note that DRP2a/2b also have functions at the plasma membrane.

Given its central position in the secretory pathway, the Golgi complex is a highly dynamic organelle with numerous incoming and outgoing transport intermediates. It has to meet a constant challenge to ensure precision in trafficking while maintain its own structure and identity. In the last two decades, increasing evidences suggest that Golgi organization is maintained by a dynamic balance of anterograde and retrograde membrane flow (Altan-Bonnet et al., 2004; Ito et al., 2014). Among those are the components of trafficking machinery, including small GTPases SAR1/Arf1/Rabs and their regulators, coat proteins, COG (conserved oligomeric Golgi) complex, soluble *N*-ethylmaleimide-sensitive fusion attachment protein receptors (SNAREs), and golgins, that operate at three intimately connected organelles, namely, ER, Golgi, and TGN. Malfunction of key proteins in the plant mutants leads to Golgi structure disruption (Table 1; Figure 1). In this review, we summarize the up-to-date knowledge on genetic evidences for Golgi maintenance in plants, with a focus on protein elements in intra-Golgi trafficking. For a detailed description and discussion on plant Golgi ultrastructure, please refer to these excellent reviews (Robinson, 2020; Kang et al., 2022).

**Table 1:** Key genetic elements involved in plant Golgi structure maintenance.

Regulators	Genes	Subcellular localization	Transport pathways	Description of abnormal Golgi structures in the mutants	References
COPII proteins	*Sar1*	ERES	ER to Golgi	• Overexpression of GTP-locked version of *Sar1* led to Golgi fragmentation and accumulation of vesicle remnants.	Faso et al. (2009), Osterrieder et al. (2010)
	*Sec24A*			• A missense mutation (R693K) in Sec24A causes Golgi bodies trapped in the globule structures, often exhibited a pronounced vesicular/tubular structure.	
P24	*P24δ3, p24δ4, p24δ5, P24δ6*	Mainly at the ER	Golgi to ER	• *p24δ3δ4δ5δ6* quadruple mutations lead to aberrant Golgi with dilated areas throughout the whole cisternae.	Montesinos et al. (2013, 2014), Pastor-Cantizano et al. (2018)
SNARE	*Sec22*	ER	ER to Golgi/Golgi to ER?	• In *sec22* mutants and *syp31syp32* pollen Golgi stacks are degenerated with much fewer and shorter cisternae.	El-Kasmi et al. (2011), Rui et al. (2021)
	*SYP31, SYP32*	Golgi	ER to Golgi/intra-Golgi		
Arf1 Arf-GEF Arf-GAP	*Arf1s*	Golgi/TGN	All known trafficking pathways	• Overexpression of GDP-locked *Arf1^T31N^* causes disintegration of Golgi stacks.	Teh and Moore (2007), Singh et al. (2018), Zhu et al. (2021)
	*GNL1*	Golgi	Golgi-ER/intra-Golgi retrograde traffic	• Knockout of *GNL1* results in Golgi stacks with laterally expanded cisternae	Richter et al. (2007)
	*AGD7*, *AGD8*, *AGD9*	Golgi	Golgi to ER/intra-Golgi, vacuolar trafficking	• Overexpression of *AGD7* or double knockdown of *AGD8* and *AGD9* causes disassembly of Golgi apparatus. In *agd8 agd9 RNAi* plant cells, only one or two cisterna is left in a stack.	Min et al. (2007, 2013)
Coatomer	*α2-COP, β-COP, β´-COP, ε-COP*	Golgi	Golgi to ER/intra-Golgi	• Disruption of *ε-, α2-,* or *β´-COP* results in defective Golgi with fewer cisternae per stack, abnormal vesicle clusters near the Golgi remnants. • Knockdown of *β-COP* causes enlarged Golgi with longer cisternae.	Ahn et al. (2015), Woo et al. (2015), Gimeno-Ferrer et al. (2017), Sanchez-Simarro et al. (2020)
COG	*COG3*, *COG6*, *COG8*	Golgi	Intra-Golgi	• In *cog3*, *cog6*, *and cog8* mutant pollen, the Golgi bodies display decreased cisternal length, increased individual cisterna and stack width.	Tan et al. (2016), Rui et al. (2020)
Putative Rab GEF	*LOT*	Golgi	TGN to vacuole/PM	• In *lot* mutant pollen, the number of TGN/EE is dramatic reduced, and number of Golgi stack is increased.	Jia et al. (2018)
Rab GTPase	*RabH1B*	Golgi	Not available	• *Rabh1b* mutation leads to abnormal Golgi and large vesicles accumulated near the *trans* side. Number of cisternae per Golgi is increased and the cisternae appear wider.	He et al. (2018)
Proteins implicated in CCVs formation	*MTV1*	TGN	Vacuolar transport	• *ADG5* mutation leads to cup-shaped or circular Golgi. • *mtv1 agd5* double mutant or *ap1m* mutants, *trans*-cisternae and TGN are bent into sickle shaped or fully circular structures.	Liljegren et al. (2009), Park et al. (2013), Sauer et al. (2013)
	*AGD5*				
	*AP1M*	TGN	TGN to vacuole/PM		
	*DRP2a*, *DRP2b*	PM/TGN	Endocytic and post-Golgi trafficking	• In *drp2a 2b* double mutant pollens, Golgi stacks are abnormal with increased number and decreased length of cisternae.	Jin et al. (2001), Backues et al. (2010), Huang et al. (2015)
SYP4s	*SYP41*, *SYP42*, *SYP43*	TGN	TGN to vacuole/PM	• In *syp42 syp43* double mutant or *tno1* mutant, *trans-*cisternae in Golgi are curved.	Uemura et al. (2012)
Putative tethering factor	*TNO1*	TGN	TGN to vacuole/PM	• In *tno1* mutant, Golgi stacks are slightly affected, association of Golgi and TGN is impaired. The *tno1* phenotypes can be rescued by overexpression of *SYP41* or *SYP61*	Kim and Bassham (2011), Yang et al. (2019)
V-ATPase	*VHA-A*	Vacuole		• In the *vha-A* bicellular pollen, aberrant Golgi structure with increased number of cisternae in a single stack, and end swollen, cup-shaped cisternae are detected.	Dettmer et al. (2005)

The Golgi phenotypes described in the table are based on the TEM micrographs.

## ER-Golgi Transport

In plants, the ER is generally juxtaposed to the Golgi due to the lack of ERGIC (ER-Golgi intermediate compartment; Hawes and Satiat-Jeunemaitre, 2005). Trafficking from ER to Golgi in plants is generally thought to be mediated by COPII vesicles (Robinson et al., 2015; Robinson, 2020), although tubes are proposed as transport carriers as well (Hawes et al., 2008; Hawes, 2012). Recently, the existence of COPII vesicles in plant is verified by an elegant cell-free reconstitution experiment utilizing materials derived from Arabidopsis cells (Li et al., 2021). The ER subdomains where COPII-coat assembly occurs is called ER exit sites (ERESs) where proteins transport to the Golgi apparatus are sequestered (Brandizzi, 2018; Weigel et al., 2021). Live-cell fluorescence in plant cells has shown that highly mobile Golgi stacks can first capture the ERESs to enable COPII-mediated cargo deliver from the ER to the *cis*-Golgi cisterna and then release ERESs for next round of cargo loading (Takagi et al., 2020). In doing so, it enables efficient ER to Golgi transport. The COPII coat consists of Sar1 and two sub-complexes, namely, Sec23/Sec24 and Sec13/Sec31 (Barlowe et al., 1994). Upon GTP binding, Sar1 exposes an N-terminal α-helix that facilitates its binding to the ER membrane, and Sar1-GTP recruits the Sec23/24 heterodimer, which forms the inner COPII coat, and then the outer Sec13/31 heterodimer assemble to forms the outer coat “cage” and deforms the membrane to generate COPII vesicles (Hutchings and Zanetti, 2019).

Sar1 activation initiates COPII coat formation. Overexpression of GTP-locked Sar1 disrupts COPII formation at the ERES, inhibiting cargo exit from the ER, at the meantime, redistributing Golgi membrane proteins to the ER (Phillipson et al., 2001; DaSilva et al., 2004; Yang et al., 2005; Osterrieder et al., 2010). In tobacco cells, expression of a dexamethasone-inducible Sar1-GTP causes Golgi bodies to gradually reduce in cisternae length and cisternae number, followed by Golgi fragmentation and accumulation of vesicles (Osterrieder et al., 2010). Sec24 has been implicated in cargo recognition (Adolf et al., 2016). A missense mutation (R693K) in Arabidopsis Sec24A induces the clustering of tubular ER and Golgi membrane, where Golgi resident and secretory proteins are accumulated. Golgi bodies trapped in the clusters often exhibited a pronounced vesicular/tubular profile. This disruption of ER-Golgi integrity could not be rescued by AtSec24B or AtSec24C complementation, suggesting nonoverlapping functions of different AtSec24 paralogs (Faso et al., 2009).

In plant, p24 proteins constitute a family of ~24 kDa type-I transmembrane proteins with cytosolic tails containing signals for packaging into COPI- and COPII-coated vesicles, and cycle between the ER and the Golgi apparatus (Langhans et al., 2008), possibly acting as cargo receptors (Montesinos et al., 2013, 2014; Pastor-Cantizano et al., 2016). The p24δ subfamily are required for COPI dependent retrograde transport of the K/HDEL receptor ERD2 from the Golgi apparatus back to the ER (Montesinos et al., 2014; Pastor-Cantizano et al., 2018). In the *p24δ3δ4δ5δ6* quadruple mutant, Golgi morphology is altered with dilated areas throughout the whole cisternae that are more prominent at the rim. Interestingly, despite the altered Golgi morphology and ERD2 accumulation in big intracellular structures, there is no obvious phenotypic alteration in *p24δ3δ4δ5δ6* mutant under standard conditions where activation of unfolded protein response (UPR) and upregulation of *Sec31A* gene might help the plant to alleviate the transport defects (Pastor-Cantizano et al., 2018).

**Figure 2:**
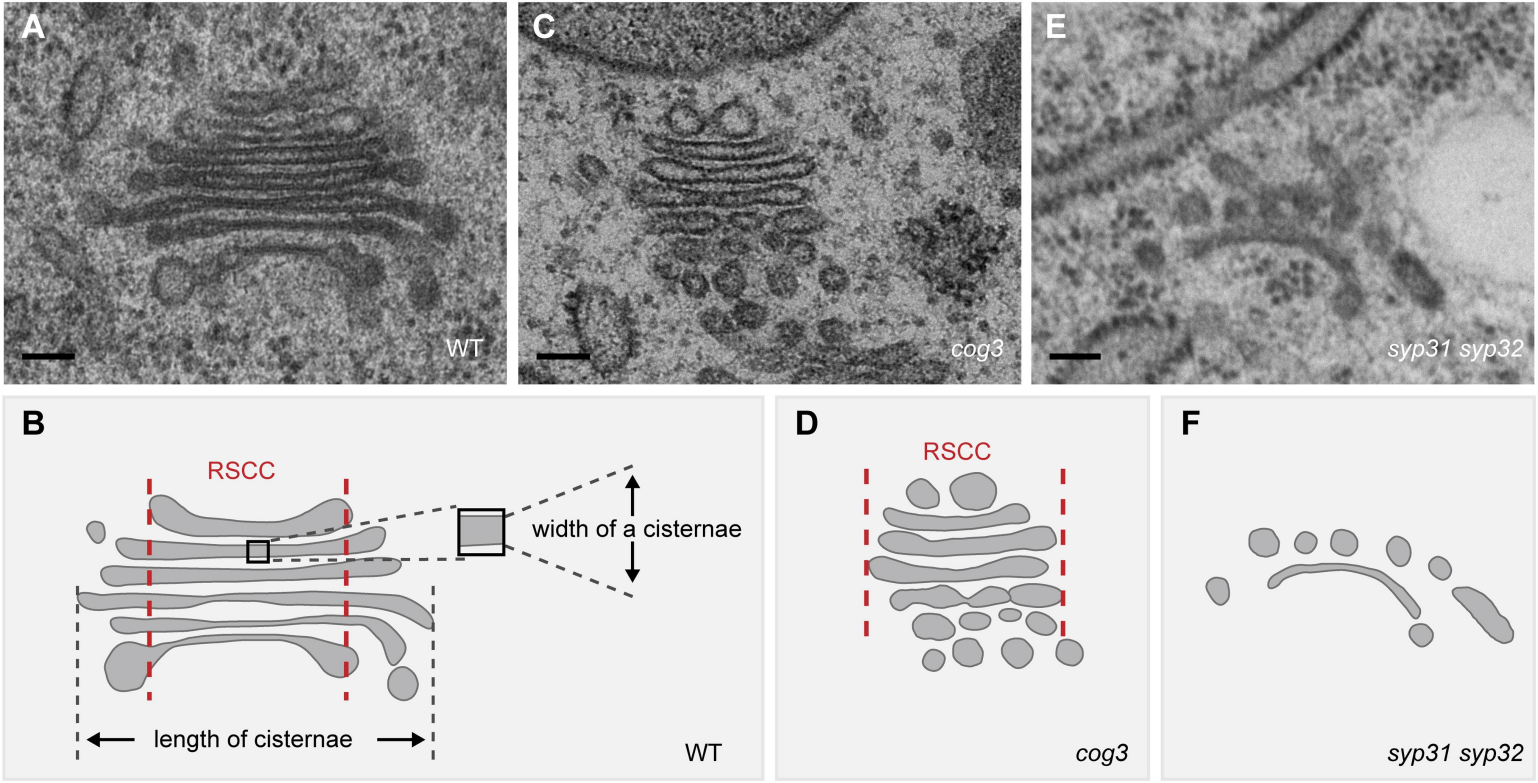
Disruption of the Golgi structure in *cog3* and *syp31 syp32* mutant pollen based on the TEM observation. **(A,C,E)** Representative TEM images of the Golgi in WT **(A)**, *cog3* **(C)**, and *syp31 syp32* **(E)** pollen. **(B,D,F)** Diagram of the Golgi structure in WT and mutants. Relative stable cisternal center (RSCC) is indicated between a pair of red lines in **(B)** and **(D)**. Bars = 100 nm.

The fusion between COPII vesicles and *cis*-Golgi membrane are mainly mediated by SNAREs, e.g., SED5/Bos1/Bet1/Sec22 in yeast (Linders et al., 2019). The function of Arabidopsis SEC22 and SED5 homologs SYP31 and SYP32 in Golgi structure maintenance has been demonstrated. In *sec22* knock-out (El-Kasmi et al., 2011) and knock-down (Guan et al., 2021) mutants, Golgi stacks appear fragmented, with a number of vesicles surrounding the cisternal remnants. A recent study from our lab demonstrates that loss function of SYP31 and SYP32, the only two Qa-SNAREs localized at plant Golgi, impairs the trafficking between ER and Golgi where mCherry-HDEL is not located to the ER, rather it is rerouted to the vacuole (Rui et al., 2021). In *syp31 syp32* pollen, Golgi stacks are degenerated, resembling that in the *sec22* mutants (Figure 2; Rui et al., 2021). Given the fact that both *sec22* and *syp31 syp32* null mutants are male gametophyte lethal and SEC22 directly interacts with SYP32 (El-Kasmi et al., 2011; Guan et al., 2021; Rui et al., 2021), SEC22 and SYP32 may function in the same SNARE complex to mediate vesicle fusion at the *cis*-Golgi. As aforementioned, these studies support that disruption of ER-Golgi transport leads to dramatic degeneration of Golgi in plant cells. Please refer to recent excellent reviews for more details in the molecular mechanisms of ER-Golgi transport in plants (Brandizzi, 2018; Pereira and Di Sansebastiano, 2021; Aniento et al., 2022).

## Intra-Golgi Transport

Retrograde transport from the *cis*-Golgi back to the ER is mediated by COPI vesicles which mediate intra-Golgi trafficking as well. In mammalian cells, COPI proteins are also involved in the anterograde transport from ER to ERGIC (Beck et al., 2009; Weigel et al., 2021). The COPI coat is composed of the GTPase ADP-ribosylation factor 1 (Arf1) and a cytosolic complex (Coatomer) made of seven subunits (α-, β-, β´-, γ-, δ-, ε-, and ζ-COP), which is recruited *en bloc* from the cytosol onto Golgi membranes (Arakel and Schwappach, 2018). COPI vesicle formation is initiated *via* the recruitment of cytosolic Arf1-GDP to the Golgi membranes by interacting with p24 family proteins (Stamnes et al., 1995; Popoff et al., 2011). Arf1 is activated by Arf1 guanine-nucleotide exchange factors (Arf1-GEFs) and the GTP/GDP exchange allows its dissociation with p24 proteins and insertion of Arf1-GTP into Golgi membranes. Coatomer can then interacts with Arf1-GTP and with the cytosolic tail of cargo proteins containing COPI-sorting signals (Popoff et al., 2011). Coatomer polymerization leads to the formation of COPI vesicles. In contrast to clathrin and COPII, COPI coat might not have discrete inner (adaptor-like) and outer (cage-like) layers (Dodonova et al., 2015).

Two morphologically distinct types of COPI vesicles, namely, COPIa and COPIb, are identified in *Arabidopsis* and algal cells (Donohoe et al., 2007). COPIa vesicles with a lightly stained content and a thicker coat mainly bud from *cis*-Golgi cisternae, and might mediate retrograde trafficking from the *cis*-Golgi. In contrast, COPIb vesicles with a dark stained content and a thinner coat bud exclusively from the *medial*- and *trans*-cisternae and are proposed to mediate intra-Golgi trafficking (Donohoe et al., 2007). Whether COPIa and COPIb vesicles contain different cargo proteins is still unknown (Gao et al., 2014; Gimeno-Ferrer et al., 2017; Sanchez-Simarro et al., 2020).

### ARF1, ARF-GEFs, and ARF-GAPs

Arabidopsis genome encodes six highly conserved Arf1 proteins share at least 97% sequence identity, suggesting their function redundancy (Xu and Scheres, 2005; Singh et al., 2018). All six Arf1s associates with Golgi/TGN (Zhu et al., 2021) and have been implicated in various trafficking steps including trafficking from the ER to the Golgi, vacuolar trafficking and endocytosis/recycling (Singh and Jurgens, 2018). The fungal toxin brefeldin A (BFA) is known as a potent inhibitor of Arf-GEFs. Short-term BFA treatment in tobacco BY-2 cells causes rapid loss of cisternae in a *cis* to *trans* direction and the loss of ARF1 and γ-COP from the Golgi membrane, indicating Arf-GEFs at the Golgi are the initial targets of BFA action in plant (Ritzenthaler et al., 2002). Overexpression of GDP-locked Arf1^T31N^ interferes with all known trafficking pathways, causing disintegration of the Golgi stacks (Singh et al., 2018), similar to what observed in BFA-treated ARF-GEF *gnl1* mutant where both GNL1 and BFA sensitive-GNOM functions are compromised (Richter et al., 2007). Disruption of Golgi-localized GNL1 results in Golgi stacks with laterally expanded cisternae, possibly due to a failure of COPI-coated vesicle formation, or induced homotypic membrane fusion between Golgi stacks (Richter et al., 2007). GNOM plays a crucial role in recycling basally localized PIN proteins and other PM cargo and is crucial for plant development (Singh and Jurgens, 2018). Besides, predominantly Golgi-localized GNOM (Naramoto et al., 2014), along with GNL1, are suggested a role in COPI-mediated Golgi-ER retrograde traffic (Richter et al., 2007). However, endogenous GNOM might play a minor role in Golgi structure maintenance because in *RNAi* (*gnl1 gnom*) seedlings, Golgi alterations detected are similar to those in *gnl1* (Richter et al., 2007), and Golgi looks largely normal in the weaker *GNOM* mutant *van7* (Naramoto et al., 2014). Arf-GAPs (Arf GTPase-activating proteins) are a group of proteins that activate the intrinsic GTPase activity of Arf, leading to hydrolysis of Arf bound GTP into GDP and dissociation of resulting Arf-GDP from the endomembrane. Arf-GAP thus regulate the duration of interaction between active Arf-GTP and its downstream effector proteins on the membrane, which further modulate various cellular processes (Singh and Jurgens, 2018). A total of 15 Arf-GAPs named Arf-GAP domain (AGD) are identified in the Arabidopsis genome, among which AGD7-AGD10 have been shown to localize to the Golgi apparatus and play roles in Golgi structure maintenance. AGD7 interacts with Arf1 and stimulates its intrinsic GTPase activity. Overexpression of *AGD7* dissociates γ-COP from the Golgi membrane, relocates Golgi protein to the ER, and impairs anterograde trafficking of vacuolar proteins. This could be explained by premature hydrolysis Arf1-GTP to Arf1-GDP, which is consistent with the finding that GDP-locked Arf1^T31N^ have the same effect (Min et al., 2007). By contrast, AGD8 and AGD9 might regulate Golgi structure and function in a GAP activity independent way by recruiting Arf1-GDP from the cytosol to the Golgi membrane. While *agd8 agd9* double knockout mutant is embryonic lethal, an inducible *agd8 agd9 RNAi* causes seedling lethal, failure to recruit γ-COP to the Golgi membrane, disassembled Golgi stack with only one or two cisternae left, and disrupted vacuolar trafficking (Min et al., 2013). Overexpression of *AGD8* and *AGD9* suppressed vacuolar trafficking defects caused by overexpression of *AGD7*, suggesting that AGD7 and AGD8/9 have complementary roles.

### Coatomer

Arabidopsis has several isoforms for each coatomer subunit except γ and δ, which are encoded by single copy gene (Bassham et al., 2008). Plant COPI vesicles could be induced, detected *in vitro*, and observed *in vivo* (Pimpl et al., 2000; Donohoe et al., 2007). RNAi knockdown of *ε-COP* expression in transgenic Arabidopsis leads to the morphological changes in the Golgi with reduced number of cisternae and a high number of vesicle clusters near the Golgi remnants (Woo et al., 2015), which is also observed upon silencing of *β´-COP* in *Nicotiana benthamiana* (Ahn et al., 2015). Very similar Golgi defects are observed in knockout mutants of *α2-COP* as well (Gimeno-Ferrer et al., 2017). However, knockdown of *β-COP* results in enlarged Golgi stacks, with cisternae significantly increased in length while the number of cisternae per Golgi seems unaffected, resembling the Golgi phenotypes caused by *gnl1* mutation (Richter et al., 2007; Sanchez-Simarro et al., 2020). However, the mechanism behind the different Golgi phenotypes upon depletion of specific COPI subunits is unclear, and further work is required.

### The COG Complex

COG, an evolutionarily conserved multi-subunit tethering complex of the CATCHR (complexes associated with tethering containing helical rods) family, coordinates retrograde trafficking within the Golgi in yeast and mammals (Ungar et al., 2006; Blackburn et al., 2019). *COG* mutations in human leads to a COG-specific typeII Congenital Disorders of Glycosylation (CDG) disease with severe symptoms where trafficking and organization of Golgi-resident glycosylation enzymes are affected (Blackburn et al., 2019). In Arabidopsis *cog3*, *cog6*, and *cog8* mutants, Golgi morphology is dramatically altered where the lengths of the cisternae are significantly reduced and concomitantly, the widths of the cisternae increased (Figure 2;Tan et al., 2016; Rui et al., 2020). The shorter cisternal length could be resulted from losing peripheral vesicles. Arabidopsis EMP12 interacts *via* its C-terminal KXD/E motif with the COPI subunit and thereby achieves steady-state *cis*-Golgi localization by retrograde transport of COPI vesicles (Gao et al., 2012, 2014). Utilizing the COPI subunit γ-COP and its cargo protein EMP12 as markers, the shortened Golgi cisternae in *cog* mutants is attributed to the loss of COPI association, thus COG might be a COPI vesicle tether in plant (Tan et al., 2016; Rui et al., 2020). These results also implicate that a plant Golgi stack might consist of a relative stable cisternal center (Figure 1, the region between two red lines) and a more dynamic cisternal margin active in COPI-mediated trafficking, supporting for the cisternal maturation model. Indeed, besides EMP12 (Gao et al., 2012), other Golgi-resident proteins involved in polysaccharides synthesis retain their localization through COPI vesicle-mediated retrograde transport (Schoberer et al., 2019a, 2019b; Hiroguchi et al., 2021), whereas cell wall polysaccharides are detected in the cisternal lumen but not in COPI vesicles (Donohoe et al., 2007). Notably, the swelling of cisternae and loosely organized stack structure, the two characteristic features of the aberrant Golgi in *cog* mutants, prompts us to speculate that COG might also play a role in inter-cisternal adhesion (Figure 2; Tan et al., 2016). In a variety of eukaryotic cells, COG subunits are considered as protein interaction hubs at the Golgi (Willett et al., 2013b), further study of the interaction network of COG might reveal new elements important for plant Golgi structure and function.

### SYP31 and SYP32

**Figure 3:**
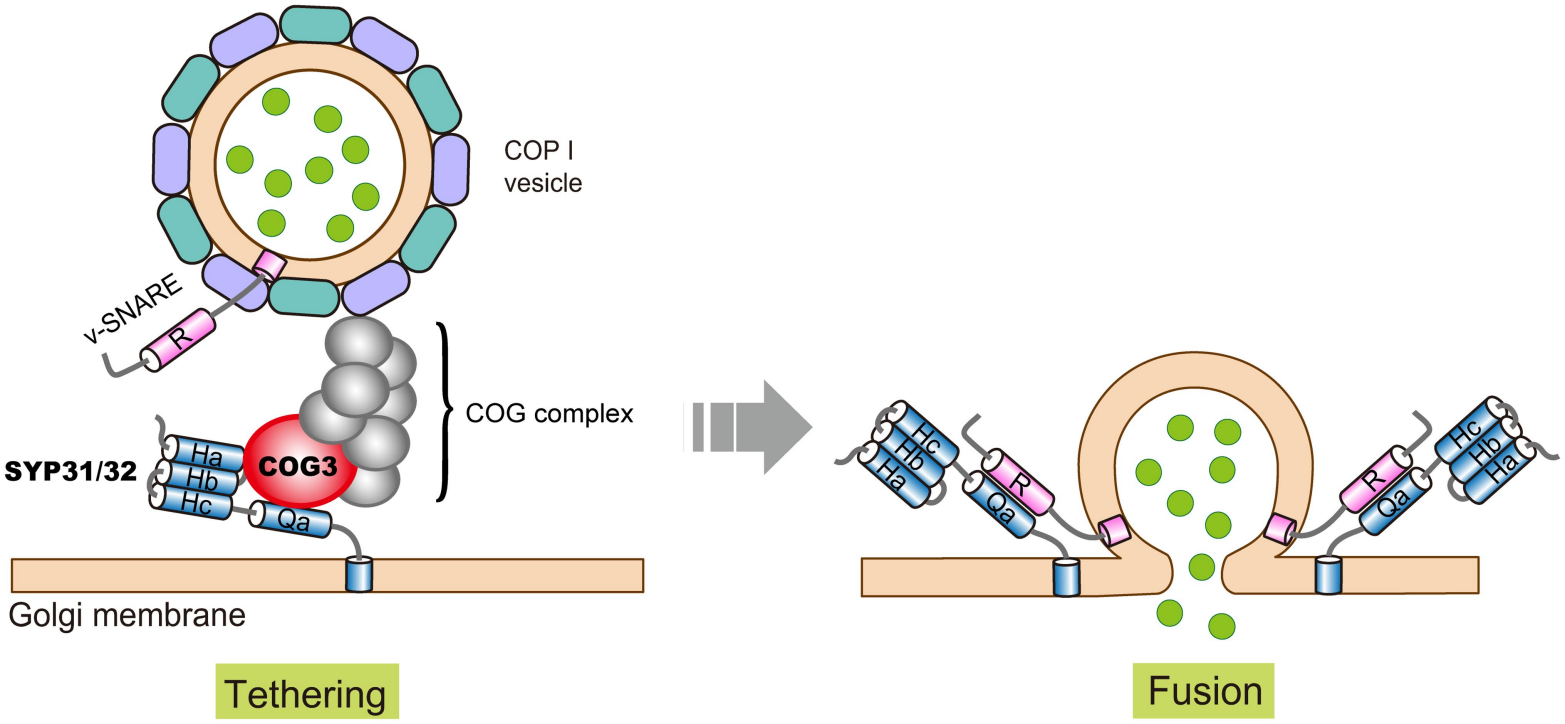
A proposed model of intra-Golgi COPI vesicle tethering and fusion in Arabidopsis. Golgi-localized Qa-SNAREs SYP31/32 recruit COG3 to the Golgi membrane, and the other COG subunits were recruited by unknown mechanisms to form a complete COG complex. The COG complex tethers the incoming COPI vesicle, followed by vesicle fusion mediated by SNARE pairing. Please note that for simplicity, Qb and Qc SNAREs are not depicted here.

As mentioned above, SYP31 and SYP32 are two Golgi localized Qa-SNAREs. In addition to their roles in ER to Golgi transport, SYP31 and SYP32 regulate COPI-dependent retrograde transport in Golgi, so as to coordinately modulate Golgi morphology during male gametophyte development. We showed that SYP31 and SYP32 co-purified with COG subunits (COG1-COG4) and recruited COG3 to the Golgi membrane by direct interaction (Figure 3; Rui et al., 2021). This is different from what happens in mammalian cells where COG subunits are thought to be recruited to the Golgi membrane by different Rabs (Blackburn et al., 2019), and COG complex might serve as landmark for SNARE proteins, as specific COG subunits can actively recruit specific SNAREs (Willett et al., 2013a, 2013b). In plant whether and how different COG subunits are recruited by different mechanisms awaits to be explored. We noticed a correlation between the order of severity in Golgi disruption and stage of developmental arrest during male gametophyte development. *Cog* pollen develops normally until pollen tube growth initiates, suggesting that Golgi disrupted to such extent can sustain for pollen development but not for tremendous surge of vesicle trafficking in rapid growing pollen tube (Tan et al., 2016; Rui et al., 2020). Whereas *syp31 syp32* pollen with degenerated Golgi arrests much earlier, i.e., during pollen mitosis I (Rui et al., 2021). Noteworthy, male transmission rate of *syp31 syp32* mutations is rescued to a largely normal level by *pSYP32:SYP32* but not by *pSYP32:SYP31* transgene, implicating functional diversities between SYP31 and SYP32 (Rui et al., 2021).

### RABH1B and LOT

Rab GTPases function as molecular switches to regulate the membrane trafficking processes including vesicle budding, transport, tethering, and fusion of vesicles (Nielsen et al., 2008; Homma et al., 2021). Mammalian Rab6 is involved in trafficking of COPI- and *trans*-Golgi clathrin-coated vesicles (CCV; Storrie et al., 2012). One of its Arabidopsis YPT6/Rab6 homologues, RabH1b localizes to Golgi and is involved in delivering CESA6 to the PM as well as in endocytosis, playing fundamental roles in cell wall formation and hypocotyl elongation. The mutation of *Rabh1b* leads to abnormal Golgi with large vesicles accumulated near its *trans* side (He et al., 2018). Arabidopsis *Loss of TGN* (*LOT*) encodes a Golgi-localized putative Rab-GEF, which can substitute a yeast GEF for YPT6 (Jia et al., 2018). Mutation in *LOT* results in defective pollen tube growth *in vivo*. Less TGN/EEs, overstacking of the Golgi cisternae and vesicles accumulation are observed in pollen, indicating that defects in TGN/EE detachment from the Golgi would increase the number of Golgi cisternae. However, if LOT function as a GEF for RabHs, and what are the RabH1b effectors responsible for Golgi structure maintenance are yet to be determined.

### Golgi Matrix Proteins

The capability for plant Golgi apparatus to keep stacking and structural integrity is especially pivotal considering it is a highly motile organelle. In animal and plant cells, the Golgi “matrix,” a ribosome-exclusion zone surrounding the Golgi, is envisaged to hold the Golgi cisternae together (Slusarewicz et al., 1994; Staehelin and Kang, 2008). Besides, inter-cisternal proteinaceous connections in plant Golgi have been observed under electron microscope (Donohoe et al., 2006; Zhang and Wang, 2015). In animal cells, two of the most prominent components of the matrix are GRASPs (Golgi ReAssembly and Stacking Proteins) and golgins. GRASP55 and GRASP65 have been implicated in the stacking of cisternae and in lateral linking of adjacent stacks into a ribbon (Jarvela and Linstedt, 2014; Zhang and Wang, 2015, 2020). Golgins are typically long coiled-coil proteins present at the cytoplasmic face of the Golgi, which mainly serve as specific tethers for distinct vesicles at the Golgi membrane (Ramirez and Lowe, 2009; Wong and Munro, 2014; Gillingham and Munro, 2016; Boncompain and Weigel, 2018).

Knockdown (by RNAi) or knockout (by CRIPSR/CAS9) of both *GRASP55* and *GRASP65* in cells leads to the disassembly of the entire Golgi stack (Xiang and Wang, 2010; Bekier et al., 2017). It has been demonstrated that GRASPs work together with golgins (e.g., GRASP65 with GM130) in Golgi stacking and Golgi ribbon formation, where GM130 initiates long-range tethering followed by oligomerization of GRASPs to form stable cross-links between apposing cisternae, thus lead to Golgi stacking (Barr et al., 1998; Lowe, 2011). However, a recent study shows that in *GRASP55* and *GRASPR65* double knockout mouse, lateral linking of the ribbon is affected while the Golgi stacks remain intact (Grond et al., 2020; Zhang and Seemann, 2021). These results leave the mechanism of Golgi cisternae stacking in animal cells an open question. Lacking of components such as GRASPs, GM130, and giantin proteins in plant cells suggests that plants may have evolved a different mechanism for Golgi stack formation.

Some putative golgins in Arabidopsis have been identified through homologue search (Latijnhouwers et al., 2005a) and studies support their roles in trafficking. Small GTPase ARL1 and Arf1 interact with golgin AtGRIP and GDAP1, respectively, on the Golgi membrane (Latijnhouwers et al., 2005b; Matheson et al., 2007). Atp115/MAG4 locates at *cis*-Golgi and mediates the transport of storage protein precursors from the ER to the Golgi (Takahashi et al., 2010). AtCASP and Atgolgin84 locates to the *cis*-Golgi and are implicated in tethering the Golgi to the ERES (Latijnhouwers et al., 2007; Osterrieder et al., 2017; Vieira et al., 2020). So far, except for Atp115, no biological study of golgin on plant development has been conducted. Also, it remains obscure whether and how golgins is involved in the stacking of cisternae.

## Post-Golgi Trafficking

Plant TGN/EE is the hub where the exocytic and endocytic pathways converge, which includes anterograde routes of secretion, vacuolar transport and recycling, and retrograde endocytic pathway. Plant TGN/EE, comprising of blebbing, tubular-reticular membranes with clathrin-coated vesicles (CCV), is a distinct organelle arises from cisternal maturation of the *trans* most Golgi cisterna (Golgi-associated TGNs, GA-TGNs), while GI-TGNs (Golgi-released independent TGNs) derived from GA-TGN are away and behave independently from the Golgi apparatus (Viotti et al., 2010; Kang et al., 2011; Uemura et al., 2014, 2019). TGNs are sub-compartmentalized molecularly and functionally (Heinze et al., 2020; Renna and Brandizzi, 2020), and most recently, two subregions of a single TGN, namely, the secretory trafficking zone and the vacuolar trafficking zone, responsible for distinct cargo sorting are proposed (Shimizu et al., 2021). Application of specific Vacuolar H^+^-ATPase (VHA-ATPase) inhibitor Concanamycin A causes the loss of TGN/EE identity possibly by increasing its lumenal pH, this leads to vacuolation of TGN/EE, Golgi cisternae bending, and Golgi fragmentation in BY-2 cells (Robinson et al., 2004). Hence, TGN integrity has profound impact on keeping normal Golgi structure.

During CCVs formation, Arf GTPases recruit adaptor protein (AP) complex, which serve as an intermediate between clathrin coat components and cargo receptors. Clathrin triskelions are recruited by AP complexes and assembled to form clathrin-coated vesicles, which are pinched from the donor membrane with the aid of the polymerizing GTPase dynamins (Jin et al., 2001; Backues et al., 2010; Law et al., 2022). AP1-4 in plants have been found to be heterotetrametric complexes, comprising two large subunits (α/γ/δ/ε and β), one medium subunit (μ) and one small subunit (σ). In Arabidopsis, several studies show that AP1, AP3, and AP4 participate in anterograde transport from the TGN to the vacuole/PM (Niihama et al., 2009; Park et al., 2013; Robinson and Pimpl, 2014; Fuji et al., 2016; Hatsugai et al., 2018; Law et al., 2022).

Molecular players implicated in CCV biogenesis at the TGN can be involved in Golgi morphology maintenance in plants. AGD5/NEV/MTV4 is a functional TGN-localized Arf1-GAP required for flower organ abscission, and mutations in *AGD5* result in cup-shaped or circular Golgi/TGN hybrid (Liljegren et al., 2009; Stefano et al., 2010). *MTV1* encodes a TGN-localized Epsin-like protein. In terms of CCV assembly, Epsins can be classified as “non-classical” monomeric adaptors. MTV1 and AGD5 interact clathrin heavy chain (CHC) and are incorporated into the CCVs, cooperating specifically in vacuolar cargo transport. In the *mtv1 agd5* double mutants, the *trans*-Golgi cisternae/TGN are bent into sickle-shaped or circular structures similar to *agd5* mutant (Sauer et al., 2013). Recently, MTV1 is shown to be recruited to the TGN by AP4 adaptor, double mutant of *espin1 mtv1* display no Golgi abnormality (Heinze et al., 2020). Thus, the abnormal Golgi structure in *mtv1 agd5* mutant might be resulted from failure of AGD5 recruitment of Arf1 to the TGN membrane and the disruption of CCV biogenesis (Sauer et al., 2013; Heinze et al., 2020). Golgi morphology is altered in a similar way upon disruption of AP1 μ-adaptin subunit AP1μ where AP1μ1 and AP1μ2 interact with AP1 γ-adaptin, mediating trafficking from the TGN to the vacuole, the PM, and the cell plate (Park et al., 2013).

Vesicle fusion and tethering at the TGN might play a role in Golgi structure maintenance. SYP41/42/43 and SYP61 are TGN-localized t-SNAREs. In *syp42 syp43* mutant, dramatically changed, curved Golgi *trans*-cisternae/TGN was observed (Uemura et al., 2012). A putative tethering factor TNO1 interacts with SYP41 and is required for correct SYP61 localization (Kim and Bassham, 2011), meanwhile, SYP41 forms a t-SNARE complex with SYP61 and VTI12 on the TGN and is required for membrane fusion (Bassham and Blatt, 2008). However, as a putative tethering factor, TNO1 only mildly affects Golgi morphology, where transport vesicles on the Golgi is reduced in *tno1* mutant, likely because of the presence of functional redundant tethers (Yang et al., 2019). In the *syp42 syp43* mutant, the abnormal Golgi structure could be attributed to arrested fusions between the *trans*-Golgi derived vesicles and the TGN, the accumulation of *trans*-Golgi–derived vesicles alters *trans*-Golgi cisternae in a curved shape. Another possibility is that the TGN function is defective, which might slowdown the *trans*-Golgi cisternae maturation processes and cause the Golgi to curl (Uemura et al., 2012). The latter might be the reason for the defective Golgi structure in the *agd5*, *mtv1 agd5*, and *ap1μ* mutants. However, the mechanism of transport between the *trans*-Golgi cisternae and the TGN remains largely unknown.

Disruption of PM- and TGN-localized dynamin-related protein (DRP) DRP2a/2b, which function in multiple trafficking pathways (Taylor, 2011; Huang et al., 2015), leads to defects in male and female gametogenesis. The *drp2a2b* double mutation causes aberrant Golgi with shorter, non-curved, increased number of cisternae and alteration of cell wall composition and structure (Backues et al., 2010). Disruption of vacuole-localized VHA-A, a member of catalytic V_1_ subcomplex of V-ATPases impaired male gametophyte development. In the *vha-A* bicellular pollen, aberrant Golgi structure with increased number of cisternae in a single stack, and end swollen, cup-shaped cisternae are detected (Dettmer et al., 2005), reminiscent of cells treated with ConcA. This is consistent with its function as a V-ATPase. However, the mechanisms underlying the roles of DRP2a/2b and VHA-A in Golgi maintenance are largely unknown.

## Future Perspectives

As an essential organelle, Golgi apparatus is involved in many aspects of plant growth and development. Yet, the research progression on plant Golgi seems relatively slow. Future investigation on plant Golgi structure maintenance will have to address a set of existing and new questions. First, what is the mechanism of Golgi transport and their cargo? What is the biochemical composition and functional significance of plant Golgi matrix? Second, is plant Golgi segregated into subdomains along its length as indicated in *cog* mutants? What is the unique composition (proteins and lipids) and function of these subdomains? Third, are there any connections between the integrity of Golgi structure and signaling events as indicated in mammalian cells (Makhoul et al., 2018)? If any, what do these events to do with plant development and stress responses? These future work will shed light on the Golgi structure and functions unique and important for plant life.

One factor restricting the Golgi research, especially the molecular mechanism of Golgi structure maintenance is the functional redundancy of expanded members in a particular protein family, such as Arf1s, SNAREs, and Rabs (Pinheiro et al., 2009; Rui et al., 2021; Zhu et al., 2021). Disruption of single gene of those families usually leads to mild or even no phenotype. To overcome the functional redundancy, high-efficiency multiplex CRISPR/Cas9 system now enables researchers to simultaneously knockout multiple genes in desired combinations (Ma et al., 2015). Since Golgi trafficking is supported by coordinated operations of different molecular machineries, analyses of interaction partners of key players should enable researchers to identify novel players in plants. Furthermore, the Golgi *trans-*, *medial-* and *cis-*cisternal specific proteomics will be very useful for identifying novel plant specific components with known cisternal localization (Parsons et al., 2019). Finally, the advance of microscopy technology will reveal great details of Golgi dynamics and structure by using SCLIM (super-resolution confocal live cell imaging) and CLEM (correlative light and electron microscopy; Nakano, 2013; Kurokawa et al., 2019).

## Author Contributions

QR, XT, FL, and YB wrote the manuscript. All authors contributed to the article and approved the submitted version.

## Funding

This work was supported by grants from the National Natural Science Foundation of China (32070190), the Natural Science Foundation of Jiangsu Province (BK20210406), and the China Postdoctoral Science Foundation (2021M701737).

## Conflict of Interest

The authors declare that the research was conducted in the absence of any commercial or financial relationships that could be construed as a potential conflict of interest.

## Publisher’s Note

All claims expressed in this article are solely those of the authors and do not necessarily represent those of their affiliated organizations, or those of the publisher, the editors and the reviewers. Any product that may be evaluated in this article, or claim that may be made by its manufacturer, is not guaranteed or endorsed by the publisher.

## Acknowledgments

We apologize to researchers whose work has not been included in this manuscript owing to space limit.
